# Making sense of intralocus and interlocus sexual conflict

**DOI:** 10.1002/ece3.4629

**Published:** 2018-12-11

**Authors:** Martijn A. Schenkel, Ido Pen, Leo W. Beukeboom, Jean‐Christophe Billeter

**Affiliations:** ^1^ Groningen Institute for Evolutionary Life Sciences University of Groningen Groningen The Netherlands

**Keywords:** natural selection, sex‐specific adaptation, sexual antagonism, sexual selection, sexually antagonistic coevolution

## Abstract

Sexual conflict occurs because males and females are exposed to different selection pressures. This can affect many aspects of female and male biology, such as physiology, behavior, genetics, and even population ecology. Its broad impact has caused widespread interest in sexual conflict. However, a key aspect of sexual conflict is often confused; it comprises two distinct forms: intralocus and interlocus sexual conflict (IASC and IRSC). Although both are caused by sex differences in selection, they operate via different proximate and ultimate mechanisms. Intralocus sexual conflict and IRSC are often not clearly defined as separate processes in the scientific literature, which impedes a proper understanding of each form as well as of their relative impact on sexual conflict. Furthermore, our current knowledge of the genetics of these phenomena is severely limited. This prevents us from empirically testing numerous theories regarding the role of these two forms of sexual conflict in evolution. Here, we clarify the distinction between IASC and IRSC, by discussing how male and female interests differ, how and when sex‐specific adaptation occurs, and how this may lead to evolutionary change. We then describe a framework for their study, focusing on how future experiments may help identify the genetics underlying these phenomena. Through this, we hope to promote a more critical reflection on IASC and IRSC as well as underline the necessity of genetic and mechanistic studies of these two phenomena.

## 1 INTRODUCTION

Males and females are often exposed to different selective pressures leading to sex‐specific adaptations, resulting in sexual dimorphism in traits such as behavior, body size, and coloration (Parker, Baker, & Smith, 1972). The symbiosis between the two sexes is a crucial component of their biology, but the fact that they are selected toward different ends means that what is adaptive to one sex can be maladaptive to the other (reviewed in Kuijper, Pen, & Weissing, 2012). Owing to this interconnectedness, sex‐specific adaptation in one sex may not occur independently of the other sex, potentially sparking an evolutionary conflict between the sexes, which can come in two distinct forms: intralocus and interlocus sexual conflict.

Intralocus and interlocus sexual conflict (abbreviated IASC and IRSC, respectively) both stem from differences in selection between the sexes but are otherwise vastly different processes. Intralocus sexual conflict occurs when males and females have optimal fitness for different genotypes at a given locus (Bonduriansky & Chenoweth, 2009; Lande, 1980; Van Doorn, 2009) and can be defined as a conflict in selection pressures experienced by alleles at this locus, causing different alleles to be favored in males and females. IRSC occurs when males and females interact (e.g., during reproduction) but the male's and the female's fitness are maximized under different conditions (Chapman, Arnqvist, Bangham, & Rowe, 2003; Parker, 1979); IRSC is therefore defined as a conflict between male and female individuals over the outcome of interactions between them.

These definitions already highlight an essential difference between IASC and IRSC. For IASC, the conflict is only apparent from a population genetics perspective, playing out on evolutionary timescales by shifts in allele frequencies in response to the tension of selective pressures acting on males and females, and therefore it has traditionally aligned to the field of evolutionary genetics (e.g., Lande, 1980; Rice, 1984). For IRSC, however, the conflict clearly takes place between individual males and females, as the outcome between interactions between them directly impacts their fitness. As such, the biological impact of IRSC is more apparent in the context of male and female reproductive behavior. Consequently, IRSC has been mainly considered from a behavioral ecology perspective in which it is treated as a specialized form of selfish behavior (e.g., Clutton‐Brock & Parker, 1995). In addition to this, many differences exist between IASC and IRSC in terms of rates of evolutionary change, how and when fitness costs occur, and how male and female biology is intertwined (see also Table 1). Despite IASC and IRSC having traditionally been considered from entirely different perspectives and the various other differences between them, they have over time been lumped together under the term “sexual conflict.”

**Table 1:** Intralocus sexual conflict and IRSC are both caused by sex differences in selective pressures. Despite this common origin, they show distinct differences in various conflict aspects involving among others fitness, adaptation, and evolution

Conflict aspect	Intralocus sexual conflict	Interlocus sexual conflict
Sexes are connected by	Shared genome/genetic architecture for shared traits	Reproduction
Males and females have optimal fitness for	Different values for a shared trait	Different reproductive scenarios
Conflict takes place over	Genetic composition of loci affecting shared trait	Outcome of reproductive interactions
Conflict takes place because	Selection favors different genotypes in males and females	Reproductive success is maximized under different conditions for males and females
Fitness is determined by	Genotype × phenotype (sex) effect	Phenotype (own) × environment (mate pool) effect
Fitness costs are imposed on affected individuals by	Inheritance	Social (reproductive) interactions
Fitness costs caused by high‐fitness individuals are imposed on	Opposite‐sex offspring	(Potential) mates
Adaptation occurs	By spread of alleles with sex‐specific benefits	By alleles that provide a benefit in the current social environment
The same genes are involved in male and female adaptation	Yes (by definition)	Typically not (different genes are assumed to affect each sex)
Potential for male‐female coevolution	Not predicted	Yes (including Red Queen dynamics)
Sexual dimorphism evolves due to	Conflict resolution	Conflict manifestation (escalation)
Timescale	Evolutionary	Ecological (outcome of interactions) to evolutionary (adaptation to social environment)
Rate of evolutionary change	Variable	Typically fast

Unfortunately, the lumping together of these two distinct phenomena can be confusing; IASC and IRSC are often not clearly distinguished from one another in the literature, making it difficult to discern which of these is discussed (Tregenza, Wedell, & Chapman, 2006; see also Box 1). Although the distinction between these two phenomena has been previously discussed (e.g., Arnqvist & Rowe, 2005; Chapman et al., 2003), the amalgamation of IASC and IRSC continues to be a source of confusion, particularly for those new to these topics or who are only familiar with IASC or IRSC. Given that IASC and IRSC can influence many processes and therefore be of interest to numerous biological disciplines, a proper understanding of these phenomena may be beneficial even to those who do not work directly on these phenomena. Therefore, it is necessary to be aware of and account for the distinction between IASC and IRSC, not only in the literature, but even more so when designing experiments or interpreting results. Without this, experimental designs may lead to confounding interpretations of the results, or we may fail to formulate a proper explanation for these results. In turn, this leads to more misunderstanding about IASC and IRSC. Furthermore, although both “intralocus” and “interlocus” emphasize the role of genes in these conflicts, we still know very little about the genes involved. Intralocus sexual conflict IASC and IRSC differ vastly in the underlying genetic architecture, and concomitantly in the relationship between genetic variation, sex, and individual fitness. This in turn results in different predictions regarding the evolutionary dynamics under IASC and IRSC. To test these theories empirically, it is essential that we learn which genes are involved in each conflict. Therefore, for future research on IASC and IRSC, it is necessary (a) to design experimental procedures that are tailored specifically to the conflict under investigation, and (b) to focus on unraveling the genetics that underlie these phenomena.

Box 1 **Pitfalls in discussing and identifying intra‐ and interlocus sexual conflict**

1 IASC and IRSC are distinct evolutionary phenomena which share a common origin in sex differences in exposure to natural and sexual selection (Table 1). Unfortunately, scientific literature is sometimes unclear or even incorrect in interpreting effects of IASC or IRSC. Despite the differences between these two phenomena, understanding the literature in which they are discussed can thus be quite challenging. Here, we outline some common pitfalls in the discussion of IASC and IRSC and provide some guidelines about how to navigate around them.

**1. Misinterpreting fitness benefits and costs**

Sexual selection acts differently on males and females, and therefore it plays a role in both IASC and IRSC. Fitness is determined differently in both conflicts, and consequently adaptation not only occurs differently in males and females, but also occurs differently under IRSC and IASC. Unfortunately, this difference is not always taken into account, resulting in observed fitness effects being misinterpreted. Under IASC, sex‐specific adaptation occurs as alleles with sex‐specific benefits spread in the population. The opposite sex experiences fitness costs when they carry these alleles. Effectively, this cost is a genetic load that an individual may inherit; this is also why IASC can cause negative heritability of fitness from one parent to its opposite‐sex offspring. Under IRSC, sex‐specific adaptation occurs as individuals of one sex evolve adaptations to the other sex. The opposite sex experiences fitness costs not because of a genetic load, but because they encounter mates to whom they have not yet adapted. This cost is thus not inherited, but imposed by the environment.

The difference in how fitness costs arise can be used to separate IASC from IRSC. Consider for example a male with a high fitness, as defined by his reproductive success. The male's high reproductive success can be, among other reasons, due to adaptation under IASC (carrying alleles with male‐specific benefits) or IRSC (utilizing a reproductive strategy that results in him successfully manipulating female mates). If it is due to IASC, then his sons should experience similar benefits, whereas his daughters should experience fitness costs from inherited alleles. In effect, fitness benefits from IASC result in fitness costs to opposite‐sex offspring. If the male has high fitness because of IRSC, then the costs are imposed on his mates. That is, the male is more effective at mate manipulation. Hence, females who mate with this male experience reduced fitness relative to females that mate with males that are lower fitness (and therefore less effective at mate manipulation). In short, fitness costs of IRSC are environmentally imposed and thus experienced by the mate of a high‐fitness individual, whereas fitness costs of IASC are imposed by inheritance and thus experienced by opposite‐sex offspring of high‐fitness individuals.

**2. Ambiguity and the misuse of sexual conflict**

IASC and IRSC are often referred to as forms of sexual conflict in a broad sense (i.e., sexual conflict then refers to the pair of these two phenomena). For example, this is used somewhat superficially to reflect on the general principle of sex differences in selective pressures. Although its superficial usage is correct, it is at times also used in a narrow sense in lieu of IASC or, more commonly, IRSC (i.e., to refer to IASC or IRSC specifically). The result is often ambiguous and blurs the lines between IASC and IRSC. Effects of IRSC or IASC are then attributed to sexual conflict in the narrow sense as used by the authors, which can create misunderstanding when they are interpreted as being attributed to “sexual conflict” in the broad sense. As a result, IASC‐derived effects could be incorrectly attributed to IRSC or vice versa. The use of sexual conflict in the narrow sense is thus often insufficiently specific. Using the terms IRSC and IASC avoids such confusion. Which of these phenomena is discussed can often be inferred from the context in which they are used, for example, by determining how said conflict affects fitness as described under (1). Nonetheless, the use of sexual conflict in the narrow sense is deprecated, and care should be taken when interpreting effects attributed to sexual conflict in general.

**3. Inconsistency in vocabulary between papers**

Building on (2), stylistic differences between papers may present another source of confusion. For example, IASC is often referred to as sexual antagonism, with genes under IASC being called sexually antagonistic genes. Likewise, a sexually antagonistic trait is a trait that is selected on differently between males and females. However, similar terms are used elsewhere to describe phenomena related to IRSC. For example, male–female coevolution under IRSC is often called sexually antagonistic coevolution, or an IRSC‐related phenotype or trait may be considered sexually antagonistic as its presence may benefit the carrier, but simultaneously be detrimental to its mates. The actual conflict that is discussed can again be inferred from the context in most instances (though often with quite some effort). For other cases, such phrases are accompanied by other errors (e.g., fitness effects are wrongly interpreted, or when sexual conflict is misused), making it increasingly hard or even impossible to properly determine which of these conflicts is being discussed. Whether or not the use of phrases such as “sexually antagonistic traits” is preferable is subject to debate, as writing style boils down to be a matter of taste. Being aware that these phrases can hold different meanings can help prevent misunderstandings; when they are encountered, it is often wise to reflect and determine which conflict is being discussed. Likewise, when using them it is often best to explicitly state the conflict they refer to in order to avoid uncertainty.

Here, we will focus on the distinction between IASC and IRSC as well as their genetic bases, by discussing (a) what intra‐ and interlocus sexual conflict entail; (b) how is fitness determined and how does sex‐specific adaptation occur under each conflict; (c) how this translates into emergent evolutionary patterns; (d) how this is achieved at the genetic level; and (e) how to design experiments to identify the genes involved. We hope to contribute to a better understanding of the rich but often confusing literature on IASC and IRSC, and to promote further study on the role of genetics in these phenomena.

## 2 INTRALOCUS SEXUAL CONFLICT

### 2.1 What is IASC?

**Figure 1:**
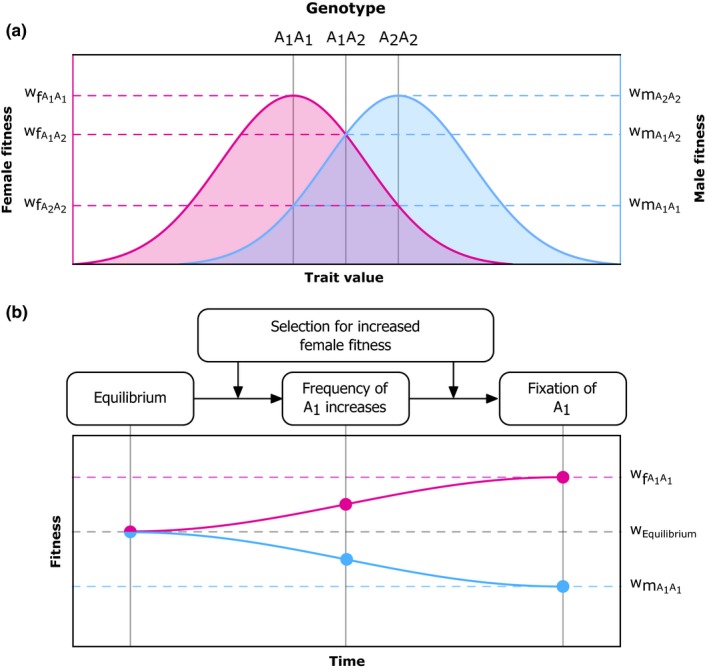
(a) Intralocus sexual conflict occurs when males and females have different optimal genotypes. Sex‐specific selection affects males and females differently, resulting in different fitness landscapes for traits between the sexes (blue and pink curves). Fitness is then maximized at different trait values in males and females. When trait values are encoded by the same gene(s) in males and females, each sex has a different optimal genotype. Here, a single locus A with alleles A_1_ and A_2_ encodes the trait value; the optimal genotype for females is A_1_A_1_, but A_2_A_2_ for males. (b) Sex‐specific adaptation under IASC leads to maladaptation in the non‐focal sex. When allele frequencies are at equilibrium, both A_1_ and A_2_ may be present in the population, leading to males and females having on average suboptimal fitness because for both sexes the optimal allele is not fixed. When the selective pressures on one sex are increased (as shown here by selection for increased female fitness), the equilibrium between A_1_ and A_2_ may be disturbed, and the female‐beneficial A_1_ allele may increase in frequency. Over time, this may lead to the fixation of A_1_, and the average female fitness (pink curve) increases (relative to the average fitness at equilibrium or unselected controls) to the optimal fitness w_FA__1A1_, while the average male fitness (blue curve) decreases to the suboptimal w_MA__1A1_ (relative to the average fitness at equilibrium or unselected controls). Note that within populations, male and female fitness components must be equal (assuming equal sex ratios), and that the changes in fitness can only be observed by comparing between, for example, populations selected for increased female fitness and control populations

Adaptive evolution occurs when selection leads to the spread of alleles that confer fitness benefits and the purging of alleles that confer fitness costs. Although natural selection may generally affect both sexes in similar ways, sexual selection affects males and females differently because of inherent differences between their sex roles. Differences in selective pressures can cause a trait possessed by both males and females (e.g., body size) to be differently affected by selection between them (Connallon & Clark, 2014). When the same gene (or genes) affects this trait in males and females, IASC occurs, because different alleles for a shared gene are selectively favored in males and females (Figure 1a). The classical example of such a trait is the body coloration of guppy fish (Fisher, 1931), with drab coloration being favored in females whereas red coloration is favored in males. Drab coloration provides a benefit to females through natural selection, as drably colored individuals experience reduced predation risk relative to red individuals (Godin & McDonough, 2003). In males, drab coloration has the same benefits, but nonetheless, red coloration is selectively favorable because females prefer to mate with red males (Brooks & Endler, 2001). Therefore, red coloration confers a net advantage, as the positive effects of sexual selection outweigh the costs imposed by natural selection. Assuming body coloration is regulated by the same genes in both sexes, this leads to IASC because alleles for drab, inconspicuous body coloration are selectively favored in females, whereas alleles for red coloration are selectively favored in males. IASC thus occurs when males and females share a genome which is selected to accommodate two different phenotypes, and consequently is a conflict over the genotype that affects the phenotype under sexually antagonistic selection.

### 2.2 Fitness and adaptation under IASC

Under IASC, the relationship between genetic variation and fitness, and therefore adaptation, differs from standard conditions, in the sense that genetic variants do not have identical effects in all individuals but rather have conflicting sex‐specific effects (Bonduriansky & Chenoweth, 2009; Lande, 1980). This has important implications for evolution through effects at the individual level (i.e., via the relationship between genotype and fitness) and by extension at the population level (i.e., via how individual fitness effects translate to the spread of alleles). On the individual level, IASC may play an important role in determining fitness, depending on the number of loci experiencing IASC as well as how strongly selection acts on these loci. An individual may experience high fitness when it carries alleles that benefit its sex, or low fitness when carrying alleles that are detrimental to its sex. To assess the role of IASC in an individual's fitness, it is necessary to determine if its genotype is associated with decreased fitness in opposite‐sex carriers (Van Doorn, 2009). This can be achieved by comparing the fitness of its same‐sex and opposite‐sex offspring. For example, a female with high fitness may carry a substantial number of female‐beneficial (male‐detrimental) alleles at loci under IASC. When transmitted to her offspring, these alleles will have positive effects on her daughters’ fitness, but negative effects on that of her sons. Relative to a female with fewer female‐beneficial alleles, the daughters produced by the high‐fitness female will exhibit higher fitness, whereas her sons will have reduced fitness when compared to the sons of a female with lower fitness. Such sex differences in the heritability of fitness have been previously described in, among others, red deer (*Cervus elaphus* L.; Foerster et al., 2007) and great tits (*Parus major* L.; Poissant, Morrissey, Gosler, Slate, & Sheldon, 2016), suggesting the existence of genetic variation with sexually antagonistic fitness effects. Similarly, Innocenti & Morrow (2010) showed that for a large number of genes in *Drosophila melanogaster* Meigen, the correlation between gene expression level and fitness may be different for males and females. This suggests that IASC may indeed occur at these loci in the form of sexually antagonistic selection on gene expression level. Although our knowledge of the identity of IASC loci remains limited, these results suggest IASC occurs across various organismal groups. As such, IASC may be a common phenomenon in sexually reproducing organisms but has remained poorly reported because in many systems it may be infeasible or even impossible to determine whether a given genotype has such negative effects in carriers of the other sex.

Under IASC, sex‐specific adaptation occurs when alleles spread in the population that are positively selected in one sex, despite being negatively selected in the other sex. As these alleles have adverse effects in the other sex, adaptive evolution in one sex can effectuate maladaptive evolution in the other sex (Lande, 1980; Rice & Chippindale, 2001). In turn, counterselection in the other sex can limit the rate of and extent to which a given allele at an IASC locus may spread. Theoretical models predict that this trade‐off between costs and benefits in the two sexes plays an important role in the spread and maintenance of alleles at IASC loci and whether or not this may result in stable polymorphism (Charlesworth, Jordan, & Charlesworth, 2014; Jordan & Charlesworth, 2012). The evolutionary dynamics at IASC loci are further influenced by various other factors, such as population structure (Connallon, 2015), reproductive systems, and the position of IASC loci in the genome (particularly with regard to genetic sex‐determining factors; Jordan & Charlesworth, 2012). Although IASC may be resolved by various evolutionary processes (further discussed below), the scope for sex‐specific adaptation via the spread of alleles with sex‐specific benefits is often constrained.

### 2.3 Separating the genetic architectures of male and female fitness is crucial to IASC resolution

Ongoing IASC is characterized by male and female phenotypes being derived from the same genetic architecture, but it may be resolved when males and female phenotypes evolve to be regulated by different genes (reviewed in Mank, 2017). Several genomic processes can contribute to such a separation. First, loci under IASC may undergo duplication, which allows for different paralogs to become expressed in males and females (Connallon & Clark, 2011; Ellegren & Parsch, 2007; Parsch & Ellegren, 2013). Following this, they may acquire a sex‐limited role, as was recently found for the gene duplicates *Artemis* (*Arts*) and *Apollo* (*Apl*) in *D. melanogaster* (VanKuren & Long, 2018), which are involved in respectively egg elongation and spermatid individualization. *Arts* and *Apl* arose by duplication some 200,000 years ago, followed by *Arts* becoming predominantly expressed in females, whereas *Apl* became active mostly in males. Moreover, both genes acquired mutations that benefit the sex in which they are expressed. Misexpression of either gene in the non‐benefitting sex results in reduced fitness, confirming that both genes are indeed sexually antagonistic. A similar but less complex solution is when IASC occurs solely over the expression level of a gene, in which case the same locus may be differentially expressed due to sex‐specific regulation. Aside from sex‐limited expression (i.e., a gene is only expressed in one sex), this may also include quantitative sex differences in gene expression levels, both of which occur abundantly in many species (Parsch & Ellegren, 2013). Although not all of these genes may have at one point been subject to IASC, it is nonetheless likely that these differences reflect to some degree sex‐specific adaptation to divergent selective pressures.

A second possibility for conflict resolution is via alternative splicing of pre‐mRNA, that may allow for males and females to derive different proteins from a shared gene (McIntyre et al., 2006). For example, alternative splicing is central to sex determination in many insects, in which genes such as *doublesex* and *fruitless* (*fru*) are commonly differently spliced in males and females (Burtis & Baker, 1989; Gailey et al., 2006; Ito et al., 1996; Ryner et al., 1996; reviewed in Geuverink & Beukeboom, 2014; Meier et al., 2013; Verhulst, Zande, & Van de Beukeboom, 2010). Some have argued that these genes were originally under sexually antagonistic selection, which has been resolved by the evolution of sex‐specific alternative splicing (e.g., Pomiankowski, Nöthiger, & Wilkins, 2004). Though this remains unconfirmed, it is clear that such genes can negatively affect fitness when not correctly regulated. For example, *fru* mRNA in *D. melanogaster* is differently spliced between males and females, yielding among others one male‐specific splice product. This encodes a protein (FRU^M^) that is essential to males as it is required for correct male neuronal development and expression of sexual behaviors (Billeter et al., 2006; Demir & Dickson, 2005; Ito et al., 1996; Ryner et al., 1996). FRU^M^ misexpression is highly deleterious in females as it induces them to express male courtship behavior and it dramatically reduces female fecundity (Demir & Dickson, 2005; Rideout, Billeter, & Goodwin, 2007). Alternative splicing of Fru pre‐mRNA allows for the correct *fru* products to be expressed in each sex. Given the deleterious effects of incorrect splicing in both sexes, *fru* is an example of a gene that may have been under intralocus sexual conflict ancestrally, which was resolved by evolving alternative splicing in males and females.

Third, genes become linked to a sex‐determining gene, which allows allele frequencies to shift between males and females. For example, a gene on the Y‐chromosome in mammals will only be transmitted to males and can therefore be selected to undergo selection for male‐benefit without repercussions for female fitness. This process has even been proposed to drive the evolution of sex chromosomes (Rice, 1987), as well as shifts in sex determination mechanisms (Muralidhar & Veller, 2018; Rice, 1986; Van Doorn & Kirkpatrick, 2007, 2010). Rice (1998) experimentally induced the effects of sex linkage on sex‐specific adaptation at IASC loci. Exploiting the genetic toolkit for *D. melanogaster*, he limited a haploid chromosome set to males for 41 generations, mimicking the inheritance pattern of Y‐chromosomes. Male carriers of this chromosome set experienced increased fitness, whereas fitness of female carriers was decreased; this suggests that male‐limited transmission resulted in the spread of male‐beneficial/female‐detrimental alleles on these chromosomes. IASC loci that become linked to a sex‐determining allele may undergo sex‐specific adaptation in a similar way.

Each of the three abovementioned mechanisms of conflict resolution results in the effect of a gene being permanently limited to one sex. This leaves such genes exposed only to the selective pressures experienced by individuals of this sex (Patten, Úbeda, & Haig, 2013), and sex‐specific adaptive evolution can occur without constraints imposed by counterselection in the other sex. The rate at which IASC can be resolved by these mechanisms, and whether specific mechanisms are favored under specific conditions are open questions with regard to IASC. To answer such questions, it is necessary to identify genes that were previously under IASC and where it was resolved, or genes that are currently under IASC (Mank, 2017).

### 2.4 Future research on IASC

Because IASC has proven difficult to detect in many systems, there has been relatively little empirical work on this subject, and theoretical considerations have dominated its study. Our lack of knowledge regarding IASC genes means testing the predictions made by theoretical models is virtually impossible. Ongoing developments in sequencing technologies may prove useful when combined with artificial selection or experimental evolution approaches.

Experimental procedures that aim to uncover the genes involved in IASC should first focus on unraveling which genes affect male fitness, which genes affect female fitness, and which affect both. This requires designing experimental procedures that can bias the rate at which sex‐specific adaptation occurs, that is, the extent to which sex‐specific selection can affect evolutionary change. For example, restricting chromosomes to one sex (sensu Rice, 1992, 1998) prevents them from being affected by selection in the opposite sex. Through this, a chromosome that is passed for example through males should become enriched for male‐beneficial (female‐detrimental) alleles. Alternatively, artificial selection for increased fitness in one sex may increase the frequency of alleles at IASC loci that benefit the focal sex (Bonduriansky & Chenoweth, 2009; Prasad, Bedhomme, Day, & Chippindale, 2007). However, this requires selection regimes tailored to fit the sex under selection, as male and female fitness are constrained by different factors and hence must be assessed differently. For both types of approaches, the role of IASC in any sex‐specific adaptation can be assessed by comparing the fitness of males and females (Figure 1b). For example, when selection pressures are skewed to allow for increased effects of female‐specific selection, then females from the selected strain should exhibit increased fitness, whereas males should exhibit decreased fitness relative to males and females from unselected control populations. Increased fitness in the focal sex combined with decreased fitness in the opposite sex indicates that sex‐specific adaptation occurred at loci under IASC. Screening strains with male‐biased and female‐biased adaptation may lead to the identification of loci that evolve differently between strains (relative to unbiased controls), which represent candidate IASC genes. Unraveling the genetic architecture of IASC is a vital step to further our understanding of the role of this conflict in causing evolutionary change.

## 3 INTERLOCUS SEXUAL CONFLICT

### 3.1 What is IRSC?

Male–female differences in potential for and limitations in reproduction lie at the root of IRSC, which can be broadly defined as the conflict between two compatible mates over the course and, by extension, the outcome of a (potential) reproductive interaction between them. Males and females can take on distinctly different roles in reproduction, and although they both benefit from reproduction by the production of offspring, they may experience different costs depending on their investment into each offspring (Chapman et al., 2003; Gowaty, 2012; Kuijper, Stewart, & Rice, 2006). Consequently, sexual reproduction requires the interaction between two parties with distinct reproductive capacities and concomitantly, distinct interests.

Classically, IRSC is considered in the context of male promiscuity versus female choosiness and is often illustrated by a female refusing to mate with a certain male, and subsequent attempts by the male to subvert the female's decision (Parker, 1979). Interlocus sexual conflict may, however, occur at any point in mate–mate interactions; conflicts may arise over, for example, the use of sperm (Birkhead & Pizzari, 2002), egg‐laying rates (Carrillo, Danielson‐François, Siemann, & Meffert, 2012; Pischedda, Stewart, Little, & Rice, 2011), female remating behavior (Parker, 2006), or parental care (Trivers, 1972). The key component in all cases of IRSC is that males and females reap optimal fitness under different, mutually exclusive outcomes. As males and females interact under IRSC, each is selected to achieve the outcome that is most favorable to themselves, even if this negatively affects the fitness of its mate.

### 3.2 IRSC as a social phenomenon in ecology and evolution

In the context of IRSC, fitness is not intrinsically determined by one's genotype, but instead is a socially influenced characteristic (Moore & Pizzari, 2005; Schneider, Atallah, & Levine, 2017). Fitness can be defined as the ability to achieve favorable outcomes in interactions with compatible mates, which can be realized either by successfully manipulating mates or by resisting manipulation by mates. Although IRSC‐related manipulations may be quite cryptic, such as when females bias sperm usage as seen in *D. melanogaster* (Lüpold et al., 2013), other manipulations are blatantly obvious, such as traumatic insemination in bed bugs (*Cimex lectularius* L.; Stutt & Siva‐Jothy, 2001). Because the classic example of IRSC is that of male promiscuity versus female choosiness, male behaviors are sometimes classified as “persistence” behaviors whereas female behaviors being labeled “resistance” behaviors (e.g., Arnqvist & Rowe, 2005). However, as mentioned above IRSC may occur over other matters as well, and in other instances it might be the female who persists in some way whereas the male will be the one resisting the female (e.g., by trying to get him to invest more time and energy in parental care). We therefore promote a different terminology; IRSC‐related behaviors in one sex are referred to as “manipulations,” and the corresponding counteractions in the other sex are referred to as “counteradaptations.” We use “manipulate” here to denote the suite of actions an individual may perform to maximize its fitness, rather than to suggest that all such actions may be subtle or sneaky. As is already implied above, the success rate of a given manipulation depends on whether the mate can resist manipulation or not (Parker, 1979). Assuming manipulations have a genetic basis (see also below), a novel successful manipulation will spread in the population—effectively, this represents sex‐specific adaptation under IRSC (Holland & Rice, 1998; Rice, 2000). As it spreads, individuals of the other sex become more frequently exposed to it and are therefore less likely to achieve a favorable outcome. Selection then favors counteradaptations in the “maladapted” sex that confer resistance to this manipulation. An individual's fitness is therefore always context‐dependent under IRSC; reaching its favored outcome depends not only on its own actions (i.e., performing a manipulation), but also on that of the mate (resisting the manipulation or not).

This principle can be extended from the individual level to cover males and females as distinct groups, in that each sex evolves in relation to the other sex and vice versa (Moore & Pizzari, 2005). The pool of individuals of one sex with which a given individual may mate (or more specifically, the manipulations utilized by them) effectively forms the selective environment to which the other sex is exposed and vice versa (Rice, 1996, 2000). When one sex evolves, this changes the selective pressures acting on the other sex to resist the novel manipulation. Male–female coevolution reflects ongoing competition between the sexes to acquire control over reproduction and is therefore often referred to as an arms race between them. The principle of male–female coevolution is perhaps most apparent in the context of two differentiating populations, in which case males and females of one population coevolve with each other, but not with those of the other population. Consider two populations A and B that are isolated from each other; females from A interact with males from A, and likewise females from B interact with males from B. Consequently, over time males and females from A become co‐adapted to each other, as do females and males from B (Parker & Partridge, 1998). When individuals from A and B meet, the low level of co‐adaptation may reveal hitherto cryptic IRSC phenotypes (Rowe, Cameron, & Day, 2003). That is, interpopulation breeding may yield markedly different numbers of offspring than intrapopulation breeding, owing to low levels of co‐adaptation between males and females. For example, reduced offspring numbers might be produced if males are unable to exploit females. This has also been suggested to represent an incipient stage of speciation, in which reproductive isolation occurs between populations due to divergent evolutionary trajectories driven by male–female coevolution (reviewed in Parker & Partridge, 1998). Inversely, manipulation by one sex may be highly successful because the other sex has not evolved to the corresponding counteradaptation. Such effects are seen, for example, in the housefly *Musca domestica* L. (Andrés & Arnqvist, 2001), in which females mated to males from other populations generally exhibited higher oviposition rates than females mated to males from their own population. As this example illustrates, the selective environment in which individuals of one sex evolve is determined by the individuals of the opposite sex that they may encounter; sex‐specific adaptation of one sex directly alters the environment to which the other sex is selected to adapt. Consequently, male–female coevolution is a cornerstone feature of the evolutionary impact of IRSC (Rice, 2000).

**Figure 2:**
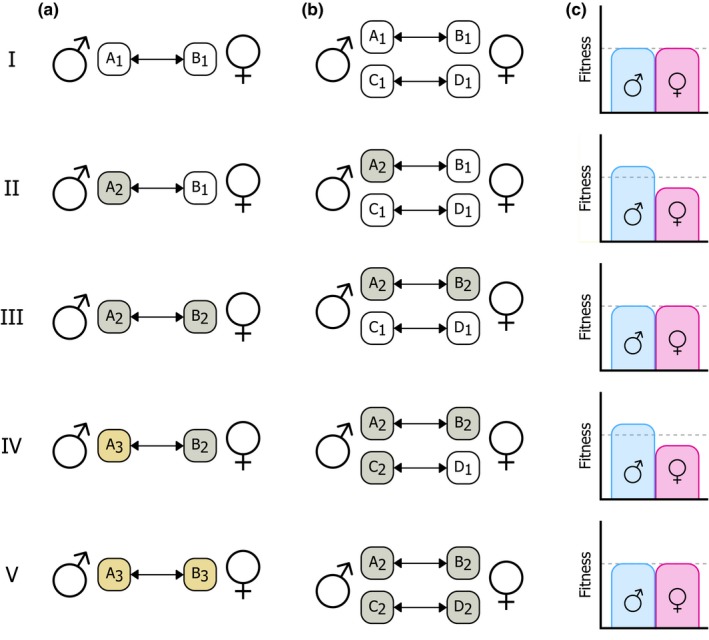
Evolutionary change under IRSC can promote ongoing diversification in different ways. When males and females have different interests in reproduction, they both may express certain phenotypes (i.e., manipulations or counteradaptations) to achieve an outcome that increases their own fitness even when this decreases the fitness of their mates. (a) Male‐female coevolution promotes ongoing change at a pair of loci which encode sex‐specific phenotypes. Here, the male and female phenotype are determined by respectively a locus A and a locus B. Invasion of a novel allele A_2_ at locus A can invoke the spread of a new allele B_2_ at locus B, which itself can cause a second new allele A_3_ to spread at locus A, Repetitions of this process can lead to alternating evolution at loci A and B. (b) New genes may acquire a role in IRSC, after which selection will favor the evolution of a correlated response in the other sex via alteration of genes underlying the interacting phenotype. Here, males and females originally express no sex‐specific phenotypes that affect IRSC, and all potential IRSC loci (A through D) are fixed for their “naïve” allele (A1 to D1, white). At some point, a new allele A_2_ at locus A spreads that confers a manipulation phenotype in males through its interaction with B in females. This triggers the spread of a counteradaptive allele B_2_ to negate the effect of A_2_. Similarly, loci C and D may eventually become involved as well when a manipulation allele spreads on C. Note that the evolutionary dynamics at the interacting loci A and B as well as C and D here are simplified, and that they may also follow those as described under (a), such that newly evolved IRSC loci may also come to exhibit ongoing turnover of alleles. (c) Fitness of males and females during coevolutionary bouts of male adaptation‐female counteradaptation

Ongoing male–female coevolution promotes novelty in two ways: by changing existing manipulations or by evolving novel manipulations altogether (Figure 2). In both cases, counteradaptations are expected to evolve to match newly evolved manipulations. When selection simply favors alteration of existing manipulations (e.g., because of IRSC being mediated in a match/mismatch type of manner), perpetual coevolution can occur between male and female traits such that they are constantly changing, which may even lead to Red Queen dynamics (for a more thorough discussion of Red Queen dynamics under IRSC, see Brockhurst et al., 2014). An alternative fate for newly evolved manipulations is that they are negated by subsequent evolution of counteradaptations. In such cases, rather than having continuous coevolution between the sexes, this instead may occur in distinct bouts. Regardless of the fate of a newly evolved manipulation, the key principle for evolutionary change under IRSC remains the same: evolution of one sex alters the selective environment to which the other sex is exposed, thereby influencing evolutionary change in this other sex as well, which together can promote continuous evolutionary change in both sexes.

Obviously, existing manipulations at one point evolved as novel manipulations (see e.g., Figure 2 in Holland & Rice, 1998). Manipulation phenotypes may evolve de novo when these exploit a pre‐existing bias (e.g., sensory) in the other sex (Arak & Enquist, 1993). Here, “bias” does not necessarily imply preference, but instead should be considered simply as a component of the mate's biology that is susceptible to manipulation. For example, *Sex peptide* (*SP*) in *D. melanogaster* encodes a seminal fluid protein that is transferred from males to females during reproduction (Chapman, Liddle, Kalb, Wolfner, & Partridge, 1995). There, it binds to *Sex peptide receptor* (*SPR*), which is expressed in the brain and in various female reproductive tissues (Yapici, Kim, Ribeiro, & Dickson, 2008). The *SP*‐*SPR* interaction affects several processes involved in the female's post‐mating response, most notably oogenesis and sperm usage (Chow, Wolfner, & Clark, 2010; Liu & Kubli, 2003); effectively, this allows males to influence female behavior. Although *SPR* was originally named for its interaction with *SP*, its presence in species lacking *SP* suggests that it may also have other functions; indeed, *SPR* interacts also with a range of myoinhibitory proteins (Kim et al., 2010). Presumably, *SP* evolved because it allowed males to manipulate female reproductive behavior, even though its receptor *SPR* was not originally involved in this component of female biology. Other IRSC phenotypes may evolve in a similar fashion, as male and female phenotypes together produce emergent pleiotropic effects on the outcome of interactions between two compatible mates.

### 3.3 IRSC is subject to constant change in its underlying genetic architectures

The potential for new manipulations to evolve and for existing phenotypes to change also leaves its marks on a genetic level. Genetic models for IRSC assume that a given locus A affects the manipulation performed by males, and a second locus B affects that of females (e.g., Rice, 2000; Rice & Holland, 1997), such as *SP* and *SPR* described above. The outcome of reproductive interactions is determined by the interaction between the female and male phenotypes. Genetic variation at A is under selection in males, with genetic variants that result in a more successful male phenotype (i.e., ones that are more likely to achieve a male‐beneficial outcome) are under positive selection, and likewise for locus B in females. A and B are commonly assumed to have no function in respectively females and males, and would therefore be selectively neutral in these sexes. Following the example of *SP* and *SPR*, we would then expect that genetic variation for *SP* is only under selection in males, and *SPR* is only under selection in females. Note that this need not necessarily be the case, and alleles that are advantageous to one sex may be disadvantageous to the other, resulting in these IRSC loci being involved also in IASC; we discuss this scenario in Box 2. Sex‐specific adaptation occurs when alleles for successful male strategies spread at locus A or when alleles for successful female strategies spread at locus B (Rice, 1998). As a consequence of male–female coevolution, sex‐specific adaptation at locus A may also trigger the spread of other alleles at locus B (e.g., Clark et al., 2009). Thus, similar to how interacting phenotypes evolve under IRSC, the genes encoding these phenotypes are also expected to coevolve.

Box 2 **Can genes be involved in both intra‐ and interlocus sexual conflict?**

1 Sex‐specific adaptation under IASC or IRSC is commonly considered only in isolation, in that typically only one of these phenomena is studied. However, both processes may involve, at least to some extent, the same genes, particularly considering that both IASC and IRSC are likely to involve traits involved in reproduction (Stewart, Pischedda, & Rice, 2010). Traits that are affected by selection as a consequence of IRSC (e.g., when a larger body size in males results in an increased ability to coerce females to mate) may also be involved in IASC (e.g., if females experience optimal fitness at smaller body sizes; Pennell & Morrow, 2013). If body size is then controlled by the same genes in both sexes, then they are involved in both IASC and IRSC. More generally, an allele with sex‐specific benefits through IRSC may be involved in IASC when it is detrimental to the fitness of opposite‐sex carriers (Table 2). Interlocus sexual conflict may then even drive the divergence in optimal trait values between the sexes, thereby sparking IASC.

**Table 2:** The strength and sign of selection acting on alleles may differ between males and females, resulting in different evolutionary trajectories

		Selective effect in females		
		+	0	−
Selective effect in males	+	Spread through natural selection	Spread through natural (sex‐specific) selection	Intralocus sexual conflict
	0	Spread through natural (sex‐specific) selection	Neutral evolution	Purged through natural selection
	−	Intralocus sexual conflict	Purged through natural selection	Purged through natural selection

Note

Positive selection in either sex results in it spreading by natural selection, whereas negative selection results in its loss. Intralocus sexual conflict occurs when both these effects occur in that an allele experiences positive selection in one sex, but negative selection in the other. When alleles are selectively neutral in both sexes, their spread or loss occurs solely through genetic drift. Alleles that increase an individual's fitness through IRSC are positively selected in at least one sex; if its influence on the fitness of opposite‐sex carriers is negative, it is also involved in IASC.

When traits are involved in both IASC and IRSC, this may also lead to different evolutionary dynamics relative to when these genes would only be involved in one of these processes (Pennell, de Haas, Morrow, & van Doorn, 2016). For example, IRSC may produce arms races between males and females which could result in escalatory evolution of the traits involved (i.e., manipulations and counteradaptations). Pennell et al. (2016) developed a model in which the outcome of male–female interactions was determined by the difference between the level of persistence exhibited by males and the level of resistance exhibited by females (similar to Rowe, Cameron, & Day, 2005), with larger differences being more beneficial to males but detrimental to females. In the absence of IASC, this model could produce a variety of outcomes, including continuous cyclical coevolution and escalatory coevolution between male and female traits, depending on factors such as the strength of selection. Under conditions that would otherwise result in escalatory evolution, Pennell et al. (2016) showed that IASC may acts as a form of balancing selection in the vicinity of evolutionary equilibria, thereby preventing male–female coevolution from causing escalatory evolution of male persistence and female resistance. Interestingly, when the outcome of IRSC was modeled based on the complementarity between male and female traits (rather than the difference between them), IASC could also prevent the system from reaching equilibria, resulting instead in cyclical patterns of coevolution in which the sign of selection acting on male and female traits switches before reaching stable values. As such, the interaction between IASC and IRSC may yield surprising dynamics depending on the mechanisms by which these phenomena affect the traits involved.

As previously discussed, IRSC can promote the evolution of novel manipulation phenotypes, which also means that genes can become involved in IRSC. Prior to the evolution of *SP*,* SPR* presumably did not play a role in IRSC in *Drosophila* spp. When *SP* arose, both it and its target receptor *SPR* became involved in IRSC. Through such evolutionary developments, IRSC might lead to the evolution of numerous such manipulations, and therefore the genetic basis of IRSC can grow more and more expansive.

As new manipulations evolve, new counteradaptations may also evolve, which can lead to two evolutionary scenarios. First, when modification of the initial manipulation allows it to subvert the counteradaptation, this can lead to ongoing coevolution between them. On the genetic level, the underlying loci for both these phenotypes tend to have high evolutionary rates (such as seen in genes with a reproductive function (Haerty et al., 2007; reviewed in Swanson & Vacquier, 2002) and may harbor higher levels of genetic variation (e.g., Hall, Lailvaux, Blows, & Brooks, 2010). Second, if variations on the initial manipulation phenotype have no such effects, then the counteradaptation simply negates the action of the manipulation. Although the rate at which either phenotype evolves may initially be high, the absence of coevolution means that the underlying genes exhibit neither the high evolutionary rates nor the elevated genetic variation. The key principles of evolutionary change in IRSC genetics are therefore that (a) diversification occurs in which genes are involved in IRSC, and (b) the involved genes can be constantly evolving (when the phenotypes they encode do so), sometimes at substantially higher rates than genes that are not involved.

### 3.4 Future research on IRSC

Owing to its potential as a driver of evolutionary change, IRSC has garnered substantial interest from evolutionary biologists. Perhaps unsurprisingly, despite multitudes of predictions on its evolutionary dynamics, we still know little about the mechanisms that mediate male–female conflicts, that is, which phenotypes are the actual manipulations or counteradaptations that are currently involved in IRSC. IRSC phenotypes may be cryptic, either in the sense that the phenotype is difficult to observe (e.g., secretion of harmful seminal fluid proteins; Birkhead & Pizzari, 2002; Chapman et al., 1995), or that the effect of a previously evolved trait in one sex is masked by correlated evolutionary responses in the other sex (e.g., when a counteradaptation negates its effects; Andrés & Arnqvist, 2001; Rowe et al., 2003). Moreover, ongoing IRSC can promote the evolution of more and diverse manipulation phenotypes, and IRSC may be mediated by multiple male and female phenotypes. For example, in *D. melanogaster*, the last male to mate with a female usually sires the majority of her offspring through last male sperm precedence (LMSP; Manier et al., 2010; reviewed in Pitnick & Hosken, 2002). Female remating reduces the proportion of offspring sired by a given male, who are thus selected to inhibit such behavior. Males have evolved several adaptations to do so, such as the transfer of SP during mating described above, but, for example, also by depositing anti‐aphrodisiac pheromones (AAPs) onto the female during copulation (Zawistowski & Richmond, 1986). These render her unattractive to other males, who are less inclined to court such a female, thereby increasing the likelihood of paternity of the first male over her offspring. Although this may benefit the male, the female's fitness is not maximized under these conditions. Recent findings indicate females may use several countermeasures to subvert male attempts to maximize their own fitness through LMSP. Females can actively shed AAPs to restore their sexual attractiveness (Laturney & Billeter, 2016). Moreover, although LMSP is extensively documented for twice‐mated females, LMSP is reduced when females mate with three or more males (Billeter, Jagadeesh, Stepek, Azanchi, & Levine, 2012). In thrice‐mated females who remated sooner (i.e., those with reduced remating latencies), the effects of LMSP were even further reduced, though these effects were not found in twice‐mated females (Laturney, Eijk, & Billeter, 2018). By altering their mating behavior, females may modulate the effects of LMSP, and therefore can have an active role in biasing the paternity among offspring instead of passively undergoing male manipulation. As these examples illustrate, the conflict over female mating behavior has led to the evolution of various male manipulations and counteradaptations in *D. melanogaster*. It also highlights the necessity for studies on IRSC to integrate various phenotypes expressed by either males or females, and to analyze if and how the expression (or lack thereof) of certain phenotypes affects opposite‐sex fitness. Although complete phenotypic surveys may be far from tractable, male and female phenotypes need to be more thoroughly investigated to understand when and how they may have a role in IRSC. Future work on IRSC should also seek to determine how these mechanisms themselves may be liable to evolutionary change. In this light, understanding the function of the multitude of reproduction‐related phenotypes displayed by males and females, and how these affect the other sex will be essential.

Considering that the IRSC phenotypes are often not fully understood, it should come as no surprise that we know even less about the genetic basis of these phenotypes. Artificial selection and experimental evolution procedures, combined with genomic and/or transcriptomic approaches, may be powerful tools to identify which genes are involved in IRSC, similar to how they may be used to do so for IASC. Here, however, the social nature of IRSC must be taken into account, as outcomes of reproductive interactions are affected by genetic variation in both males and females alike (Schneider et al., 2017). This calls for approaches in which variation in both sexes is explicitly included in studies (e.g., Chow et al., 2010), but can also be achieved using approaches in which genetic variation in one sex is artificially negated (e.g., Rice, 1998). Following the process of sex‐specific adaptation, genomic and transcriptomic sequencing can be applied to identify candidate loci that show signs of adaptive evolution. Confirming the involvement of these loci in sex‐specific adaptation under IRSC requires not only demonstrating that they increase fitness of the bearer, but also that they decrease the fitness of its mates. Both these fitness effects may, however, be context‐dependent, and therefore it is necessary to first test for these effects in conditions under which sex‐specific adaptation took place (i.e., by testing focal individuals together with mates from the non‐coevolving population). Similarly, it remains to be tested whether sex‐specific adaptation under IRSC is prone to occur at the same loci, or whether different replicate experiments may yield substantially different results. Altogether, future research on IRSC should be aimed at (a) identifying which phenotypes play a role in male–female interactions, (b) determining how these phenotypes are encoded on a genetic level, and (c) understanding how the social environment drives evolutionary change in these genes.

## 4 CONCLUSION

The observation that males and females are differently selected upon can result in two evolutionary conflicts between the sexes: IASC and IRSC. Although they are both caused by conflicting sex‐specific selection, adaptation to these selective pressures occurs differently under IASC than under IRSC. Consequently, the resulting evolutionary dynamics, as well as the impact on the genomic level, also differ between them. Here, we have discussed IASC and IRSC, and the differences between them, to promote a more critical reflection on these phenomena in past, present, and future work. We present this clarification to enable others to make sense of these two forms of sexual conflict.

Theoretical explorations of IASC and IRSC have yielded a variety of predictions on their evolutionary dynamics. Testing these hypotheses will require in‐depth knowledge of the genes involved in the conflict under investigation. Determining how genetic variation in these genes relates to differences in fitness will be crucial to understanding when, how, and at what rate sex‐specific adaptation occurs. Evolve‐and‐resequence approaches in which sex‐specific adaptation is experimentally enabled may provide insights into the identity of genes involved in either IASC and/or IRSC. However, given the different conditions under which sex‐specific adaptation take place in these conflicts, care should be taken to discern between them in these procedures as to prevent confounding effects. In this regard, the difference in which fitness costs are manifest under IASC and IRSC makes it possible to distinguish between them to assess their role in sex‐specific adaptation.

Although sexual selection has been studied predominantly in species with separate sexes, the potential for and impact of these conflicts in other mating systems (i.e., involving hermaphrodites) receives substantially less attention, even though early work on sexual conflicts in such systems (e.g., Charnov, 1979) may have laid the groundwork for research on this subject in gonochorists (Schärer & Janicke, 2009). Gonochorism, in which individuals develop into either a male or a female, does by far not apply to all sexual organisms; instead, hermaphroditism and reproductive modes in which hermaphrodites coexist along males and/or females are commonly found in many large and important organismal groups such as plants, algae, and lower metazoans (Bachtrog et al., 2014; Beukeboom & Perrin, 2014). Both IASC and IRSC may have a profound evolutionary impact in such species (see also Box 3).

Box 3 **The scope for IASC and IRSC in different reproductive systems**

1 Intralocus sexual conflict and IRSC are evolutionary conflicts between males and females and are commonly discussed in the context of gonochorism (or dioecy in plants). Many species are not gonochoristic (Bachtrog et al., 2014; Beukeboom & Perrin, 2014), and instead one individual may exhibit both male and female functionality, that is, simultaneous or sequential hermaphrodites. Aside from pure gonochorism and pure hermaphroditism, hermaphrodites and males and/or females may coexist to form yet other reproductive systems such as gynodioecy (Bachtrog et al., 2014; Charlesworth & Charlesworth, 1978). Although male and female reproductive functions can co‐occur in one individual, this does not mean that IASC and IRSC are absent in non‐gonochoristic species (Bedhomme et al., 2009). Instead, it is necessary to reconsider both as conflicts between male and female functions which may or may not be isolated from one another (Schärer, 2017; Schärer & Pen, 2013). To understand the role of IASC and IRSC in non‐gonochoristic species, it is crucial to consider how these processes may involve hermaphrodites, and how their presence may influence the dynamics of IASC and IRSC (Schärer, Janicke, & Ramm, 2014).

**Intralocus sexual conflict**

In hermaphrodites, a single genotype (and for simultaneous hermaphrodites, a single phenotype) needs to accommodate both the male and female sex function in one individual. Intralocus sexual conflict in hermaphrodites occurs when the male and female fitness components are maximized for different genotypes (sensu Figure 1). In effect, this resembles a genetic constraint, in which alleles with positive effects on male reproductive function are associated with decreased female function or vice versa (Jordan & Connallon, 2014; Olito, 2016). On a population level, stable polymorphism at IASC loci may occur, with both male‐beneficial/female‐detrimental and male‐detrimental/female‐beneficial alleles persisting in hermaphroditic populations (Jordan & Connallon, 2014; Olito, 2016). Effectively, some hermaphrodites then are “better males,” whereas others are “better females.” If and when such effects may favor the evolution of gonochorism (sensu Charlesworth & Charlesworth, 1978) remains unanswered (Van Velzen, Schärer, & Pen, 2009).

Intralocus sexual conflict in hermaphroditic systems is steadily attracting interest, but it has not yet been considered extensively in complex mating systems in which hermaphrodites coexist with males and/or females. Such systems may allow us to determine whether loci that affect trade‐offs between female and male functions of hermaphrodites are also under sexually antagonistic selection in male and female individuals (and vice versa). If so, then an allele that, for example, increases the fitness of females but decreases that of males should likewise increase female fitness component in hermaphrodites while decreasing the male fitness component. Selection in females may then promote the spread of such alleles in, for example, a gynodioecious species, despite the negative effects on the male fitness component of hermaphrodites. Inversely, alleles that increase the male fitness component of hermaphrodites may spread due to similar effects, despite decreasing fitness in female carriers. The evolutionary dynamics of IASC loci in such systems relative to pure gonochoristic or hermaphroditic systems remains a topic of future research.

**Intralocus sexual conflict**

**Figure 3:**
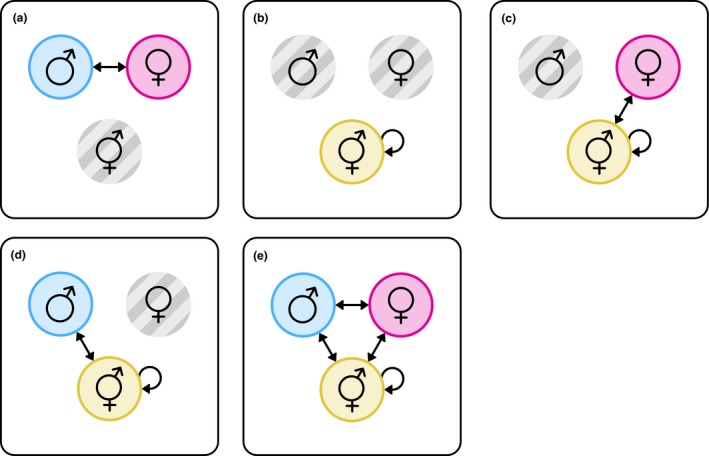
Possible reproductive interactions under different reproductive systems. (a) Gonochorism; (b) hermaphroditism; (c) gynodioecy; (d) androdioecy; (e) trioecy. Colored circles indicate the presence of that sex in the reproductive system; hashed gray circles indicate absence; arrows indicate mate compatibility between sexes

Like with gonochorists, IRSC in non‐gonochoristic species occurs between two compatible mates whose fitness is maximized for different reproductive scenarios. Hermaphrodites can interact with males due to their female function (and vice versa), with females due to their male function, and with other hermaphrodites via both sex functions. In this light, it is better to think of IRSC as the conflict which occurs because the individual's male fitness component and the female fitness component of its compatible mate are maximized under different conditions. Different reproductive systems allow for different compatible mating pairs to be formed (Figure 3); hermaphrodites are, however, compatible with all other individuals, which allows them to engage in more types of interactions than single‐sex individuals.

Despite this difference, hermaphrodites are still subject to the same principles as gonochorists, in that the pool of compatible mates shapes the selective pressures to which a hermaphrodite is exposed (Beekman, Nieuwenhuis, Ortiz‐Barrientos, Evans, & Beekman, 2016). Through the female fitness component, they are affected by interactions with individuals exhibiting the male sex function (males and other hermaphrodites), and they are similarly affected via the male fitness component by individuals exhibiting the female function. Additionally, the dual potential of hermaphrodites may also be subject to conflict, as a hermaphrodite's allocation to the male and female sex role might be manipulated by its mates (e.g., Marie‐Orleach et al., 2017). As oogenesis is energetically more costly than spermatogenesis per gamete, hermaphrodites may seek to increase their mates’ egg production (or more generally when one sex role is more costly than the other; Bedhomme et al., 2009). Inhibiting the male reproductive function may provide similar benefits by reducing “male–male” competition in hermaphrodites (Beekman et al., 2016). This is seen in, for example, the great pond snail *Linnaean stagnalis* L., which transfer seminal fluid proteins (SFPs) during copulation. Exposure to SFPs in *L. stagnalis* reduces paternity success in subsequent mating attempts (Nakadera et al., 2014). Hermaphrodites may also be choosier as to whose sperm they use to fertilize their eggs. That is, they willingly transfer sperm to other hermaphrodites while refusing to accept sperm from their mates. Coevolutionary patterns such as observed in gonochorists are likely to occur in non‐gonochorists as well, in the sense that adaptations that are beneficial to one sex function may evolve, and counteradaptations that benefit the other sex function arise in response. However, the different number and types of interactions that may take place in non‐gonochoristic species may lead to yet other patterns of adaptive evolution as the embodiment of and relationship between these coevolving functions differs from gonochorists.

As mentioned at the outset, sexual dimorphism is typically considered as a consequence of sex‐specific adaptation. Such adaptations may arise through both IASC and IRSC. Under the former, sexual dimorphism is a result of conflict resolution, whereas under the latter it is due to conflict manifestation. Its absence should not be interpreted as proof of absence of IASC or IRSC. Rather, it is possible that the former may have not yet been resolved, and the latter may not have yet developed. Developing research questions to investigate to what extent and at what rate both processes contribute to the evolution of sexual dimorphism will benefit from a thorough consideration of what IASC and IRSC actually entail. The key hurdle to achieving this will be the ability to account for the subtle differences in the origin of selection, and consequently the nature of sex‐specific adaptation under both processes.

## CONFLICT OF INTEREST

None declared.

## AUTHOR CONTRIBUTIONS

MAS, IP, LWB, and J‐CB conceived the manuscript; MAS wrote the initial draft of the manuscript; MAS, IP, LWB, and J‐CB all commented on later versions of the manuscript.

## DATA ACCESSIBILITY

There is no data to deposit for this manuscript.
